# Comparison of femoral neck system versus cannulated cancellous screws for the fixation of femoral neck fracture: a single-center retrospective cohort study

**DOI:** 10.1007/s00590-024-04051-0

**Published:** 2024-07-31

**Authors:** A. Caldaria, E. Gambuti, N. Biagi, E. Spadoni, A. Saracco, L. Massari, G. Caruso

**Affiliations:** 1 Department of Orthopedic and Trauma Surgery, San Pietro Fatebenefratelli Hospital, 00189 Rome, Italy; 2 Faculty of Medicine and Surgery, UniCamillus-Saint Camillus Internation University of Health and Medical Science, 00131 Rome, Italy; 3 Department of Neurosciences and Rehabilitation, University of Ferrara, Ferrara, Italy; 4 Orthopaedics and Traumatology Unit, S. Anna University Hospital of Ferrara, Ferrara, Italy; 5 Henley Business School, Business Informatics System and Accounting, Information Research Centre, University of Reading, Reading, UK

**Keywords:** Femoral neck fracture, Cannulated cancellous screws, Femoral neck system, Internal fixation

## Abstract

**Introduction:**

The dynamic hip screw (DHS) and cannulated compression screws (CCS) have been the two implants most frequently employed for the fixation of femoral neck fractures. The objective of this study is to compare clinical and radiographic outcomes between patients treated with the FNS and those treated with CCS.

**Material and methods:**

We conducted a retrospective analysis of a consecutive series of femoral neck fractures treated with FNS or CCS between May 2019 and June 2022. The study included 144 patients who met the inclusion criteria. Collected data encompassed age, sex, Garden fracture classification, Pauwels classification, duration of surgery, length of hospital stays, Harris Hip Score (HHS), complications, and injured side.

**Results:**

The FNS group comprised 70 patients, while the CCS group consisted of 74 patients. The operation time was 43.6 ± 12.09 min for the FNS group and 56.47 ± 22.42 min for the CCS group. At the end of the follow-up, the mean HHS was 87.07 ± 11.77 for the FNS group and 76.20 ± 13.64 for the CCS group. The mean reduction in hemoglobin levels from pre- to post-surgery was 1.05 mg/dl in the FNS group and 0.87 mg/dl in the CCS group. The reintervention rate was 8.1% for the CCS group and 2.85% for the FNS group.

**Conclusion:**

The FNS does not demonstrate superiority over CCS regarding femoral neck shortening, complication rate, and reduction in hemoglobin levels. However, FNS does appear superior to CCS in terms HHS, operation time, and reoperation rate.

## Introduction

Incidence of hip fractures has demonstrated a declining trend in the majority of countries in the last decades [1]. This favorable pattern is attributed to enhanced post-hip fracture care, implementation of fall prevention programs, advancements in lifestyle factors (including decreased rates of smoking and alcohol consumption), higher body mass index, and greater utilization of calcium and vitamin D [2–4]. Nevertheless, the diminishing trend in hip fractures has shown signs of slowing down or stabilizing in recent years across several countries [5]. Despite the decreasing trends observed in hip fracture incidence, projections indicate a significant increase in the number of hip fractures by the year 2050. According to the World Health Organization (WHO), the global population aged 85 and above is estimated to grow by 4.5 times from 2010 to 2050 [6]. Consequently, the declines in hip fracture incidence observed in many countries may not be adequate to counterbalance the effects of population aging and the consequent improved risk of hip fractures among older individuals. For this reason, hip fractures continue to represent a significant global public health concern due to their associated morbidity and mortality rates. The femoral neck fractures represent approximately 50% of all hip fractures [7], largely due to the femoral neck is a critical site for stress concentration.

Treatment approaches vary depending on the patient's age and the type of fracture. According to the American Academy of Orthopaedic Surgeons Evidence-Based Guideline on Management of Hip Fractures in the Elderly [8], there is moderate evidence supporting operative fixation for patients with stable (nondisplaced) femoral neck fractures. Indeed, in many cases involving young patients, internal fixation is the preferred primary treatment option. Cannulated compression screws (CCS) are currently the most utilized device in clinical practice. The femoral neck system (FNS; DePuy Synthes, Johnson & Johnson Medical Devices, New Brunswick, NJ, USA) is a new device launched in 2018. The efficacy of the FNS design lies in its screw-plate construct, which facilitates stronger fixation. Additionally, the incorporation of a combination of blade and anti-rotation screw enhances both axial and rotational stability. In recent years, numerous articles have compared clinical outcomes in patients treated with FNS to those treated with CCS. However, the data appear to be divergent, particularly concerning intraoperative blood loss, postoperative Harris Hip Score (HHS), operative time, fracture healing time, and postoperative complications [9–13].

Currently, there are no concrete recommendations regarding the type of device to be used in femoral neck fixation. The aim of this study is to compare clinical outcomes in patients treated with FNS and in patients treated with three CCS. Additionally, particular attention was directed toward parameters for which conflicting data persist in the literature.

## Material and methods

A retrospective analysis of a consecutive series of femoral neck fractures treated between May 2019 and June 2022 was carried out at the Orthopedics Department in the Arcispedale Sant'Anna of Ferrara (Italy).

Inclusion criteria were: isolated femoral neck fractures, closed reduction and internal fixation surgery performed with FNS or three CCS, minimum clinical and radiographs follow-up of 12 months, no preexisting decreased mobility or severe hip diseases.

Exclusion criteria were: need to perform open reduction, pathological or atypical fractures, patients with polytrauma or life-threatening clinical conditions, closed reduction and internal fixation surgery performed with two CCS, patients with cognitive dysfunction or mental disorders, unsatisfactory fracture reduction, patients undergoing prosthetic replacement.

The surgeons who performed the surgeries are trauma surgeons with at least five years of experience.

The Garden alignment index was employed to assess the adequacy of reduction [14]. Collected data included age, sex, Garden fracture classification, Pauwels classification, duration of the surgery, length of hospital stays, Harris Hip Score (HHS), complications, and side of injured were meticulously recognized. All patients received follow-up appointments at 6 weeks, 3 months, 6 months, and 12 months following their surgeries. Postoperatively, all patients underwent anterior–posterior (AP) and lateral (LP) hip radiographs. Subsequently, they were assessed and measured using the picture archiving and communication system (PACS).

**Fig. 1:**
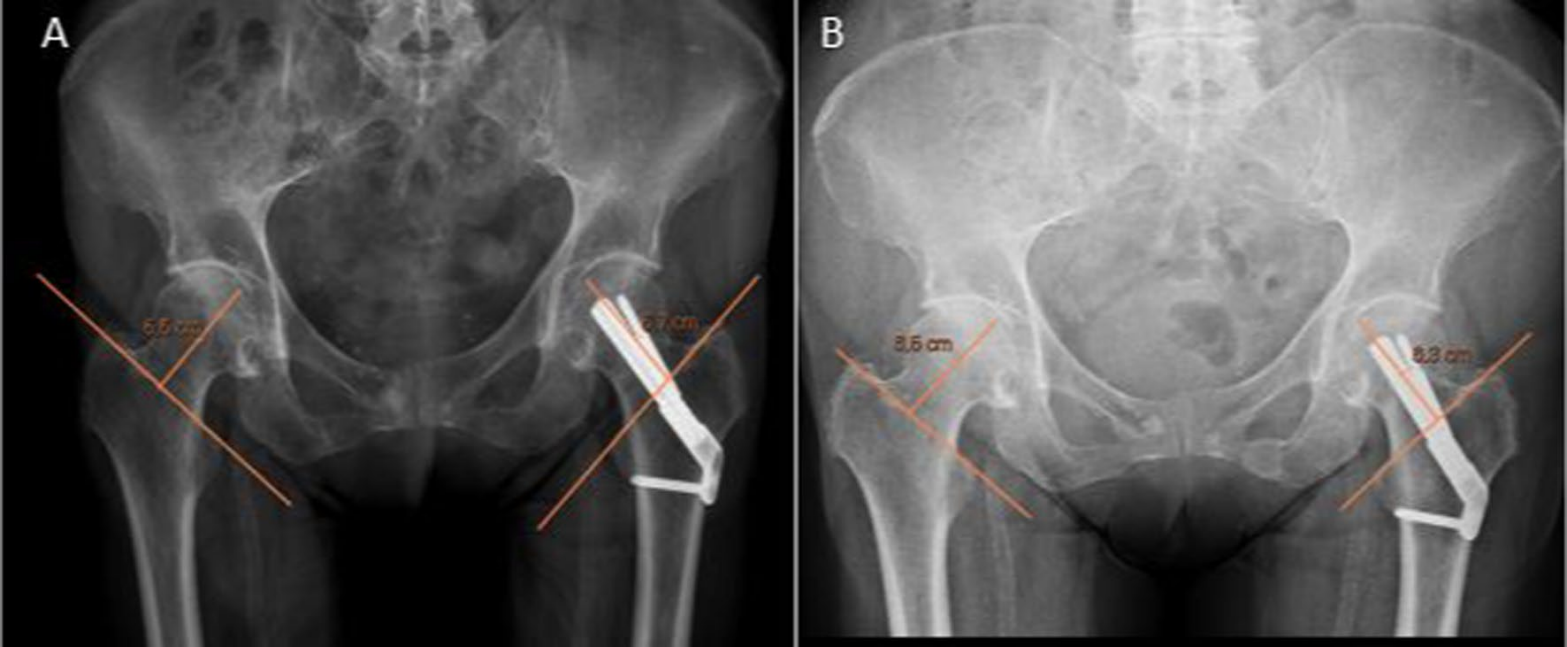
Method for measuring the shortening of the femoral head as described by Yin et al.

The shortening of the femoral head was assessed via standard X-ray using the methodology outlined by Yin et al. [15]. The shortening of the femoral head is determined by drawing a line parallel to the neck axis, starting at the intertrochanteric line, and extending through the center of the femoral head to its tip. This is done on both the fractured and the contralateral side (Fig. 1).

## Surgical technique

All patients received a preoperative dose of cefazolin sodium (2 g intravenously) or clindamycin (600 mg intravenously) in cases of beta-lactam allergy. Anti-thrombotic prophylaxis with low-molecular-weight heparin was administered before surgery and continued until walking resumed, and in all cases for a minimum of 30 days. Patients underwent treatment under spinal or general anesthesia and were positioned in a supine position on a fracture table. The procedure involved utilizing a fracture table for closed reduction, guided by G-arm fluoroscopy.

FNS was implanted based on standardized procedures according to the manufacturer’s technical manuals: Perform a skin incision approximately 6 cm in length, beginning 2–3 cm proximal to the center of the femoral neck axis. Adequately expose the lateral femoral surface to facilitate good device position. Insert guide wire, ensuring it is centrally positioned in the femoral neck both in the AP and lateral view, with the tip located 5 mm away from the subchondral bone. To prevent inadvertent rotation of the femoral head, insert an additional unused wire as an anti-rotation wire into the superior/anterior part of the femoral neck. Measure the length and carefully ream the dense bone. Proceed to manually insert the implant over the central guide wire. Subsequently, insert the anti-rotation screw based on the selected construct size. Insert the locking screw with the determined length, as indicated by the drill bit or depth gauge reading. After confirming satisfactory fracture reduction and internal fixation position using fluoroscopy, remove the anti-rotation wire and proceed to suture the wound.

Instead, patients treated with CCS received three 16 mm threaded 7 mm CCS in inverted triangle configuration. Typically, we initiate by inserting the guide wire for the lower screw. The guide pin should be positioned as inferiorly as possible along the calcar, ensuring placement in the lower region of the femoral head in the AP view, and in the center of the head in the lateral view. Subsequently, two additional guide pins are positioned, with both placed centrally in the AP view. In the lateral view, one guide wire is placed anteriorly and the other posteriorly.

## Statistical analysis

Statistical analyses were conducted using R (R Core Team, 2020), RStudio (RStudio Team, 2020), and the rstatix package (Kassambara, 2023). In all test, statistical significance was considered for *P* < 0.05. Normal distribution was determined by the Shapiro–Wilk test. For comparison between groups, the unpaired Student *t* test or nonparametric Wilcoxon singed-rank test was used for continuous variables, and the *X2* test, Fisher exact test for categorical variables.

## Results

**Fig. 2:**
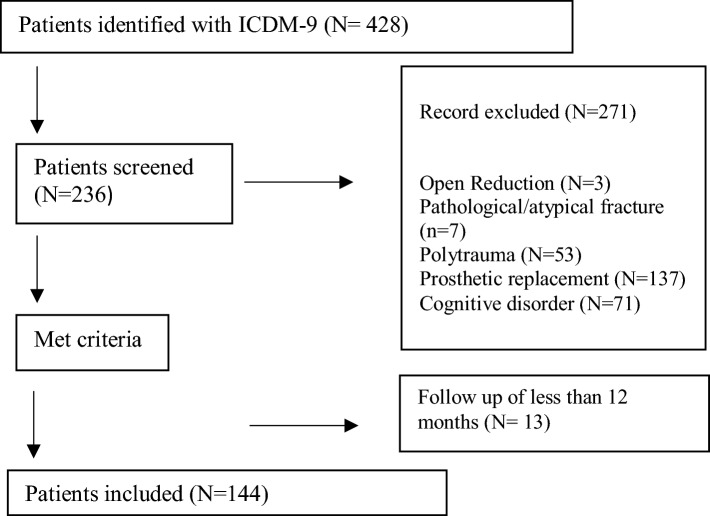
Study flowchart

A total of 144 patients with femoral neck were included in this retrospective study (Fig. 2).

**Fig. 3:**
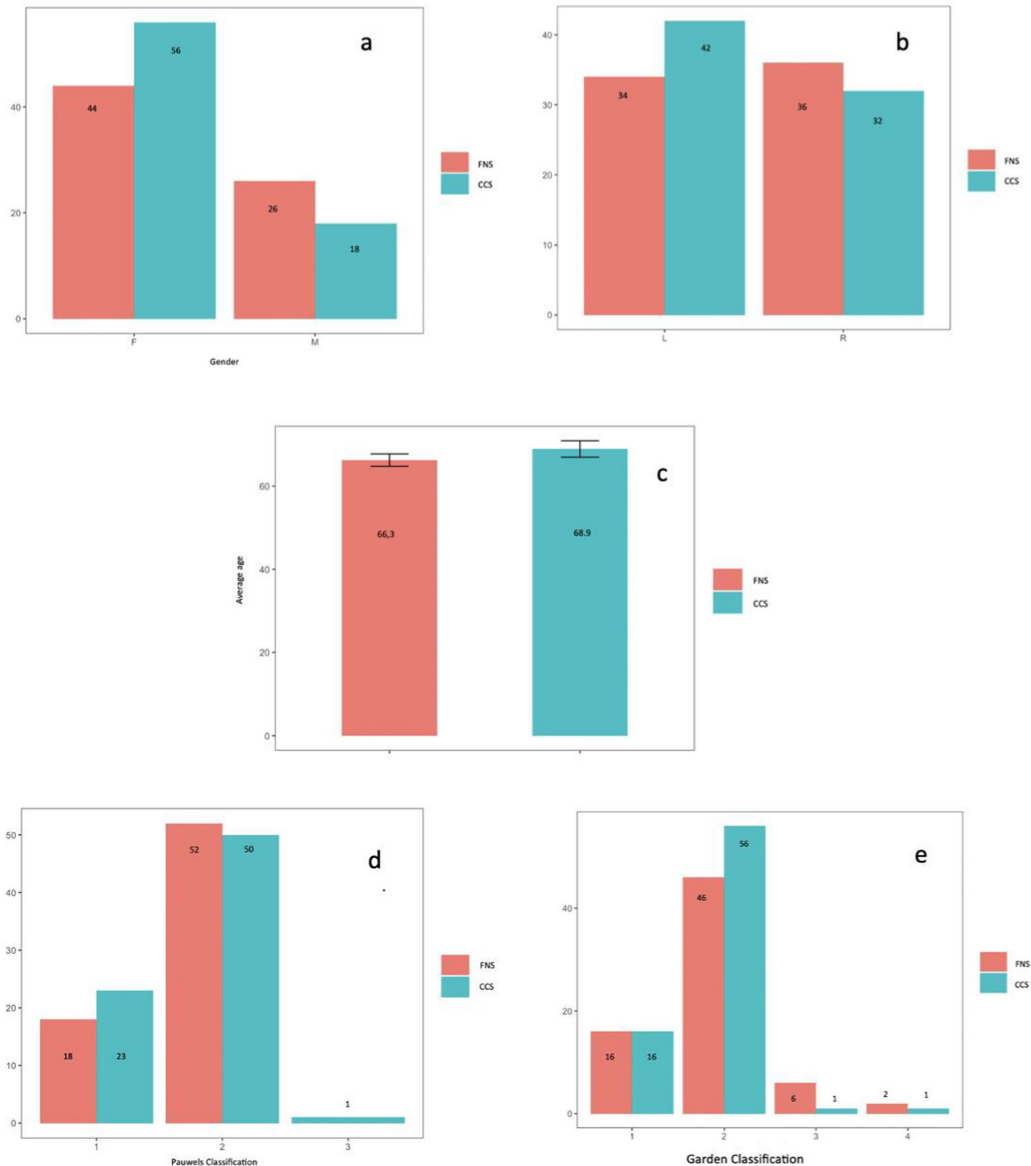
Bar plot for difference between FNS and CCS in terms of Gender **a** (*p* = 0.09), laterality **b** (*p* = 0.96), average age **c** (*p* = 0.07), Pauwels classification d (*p* = 0.46), Garden classification **e** (*p* = 0.19)

The FNS group and the CCS group, respectively, included 70 patients and 74 patients. The demographics, Garden fracture classification, and Pauwels classification are shown in Fig. 3. No significant differences were found between the two groups. The Garden classification type II and Pauwels classification type II were the most encountered in both groups. The mean length of stay was 10.3 days for FNS group and 8.6 days for CCS group (*p* = 0.01). The mean operation time was significantly shorter in FNS group (43.6 ± 12.09 min) than in CCS group (56.47 ± 22.42 min) (*p* = 0.01), with a small effect size (*r* = 0.2). The evaluation of the HHS demonstrated a significant difference between FNS group (87.07 ± 11.77) and CCS group (76.20 ± 13.64) (*p* < 0.01) with a moderate effect size (*r* = 0.39). There was no significant difference in terms of femoral head shortening (*p* = 0.87) and quality of reduction (*p* = 0.07). The mean reduction in hemoglobin levels from pre- to post-surgery was 1.05 mg/dl in the FNS group and 0.87 mg/dl in the CCS group. However, this variance did not demonstrate statistical significance (*p* = 0.86). No statistically significant variations were observed in either group for the change in hemoglobin levels from pre-surgery to discharge (*p* = 0.19), red blood cell counts (RBC) from pre- to post-surgery (*p* = 0.77), or hematocrit (HCT) from pre- to post-surgery (*p* = 0.98). In the FNS group, one patient developed femoral head necrosis, three patients reported persistent inguinal pain without signs of femoral head necrosis, and one patient experienced failure with screw back out. Patients with femoral head necrosis and screw back out underwent surgical intervention involving implant removal and prosthetic replacement.

In the CCS group, two patients developed femoral head necrosis, while four experienced failure with screw back out. All these patients underwent surgical treatment with implant removal and prosthetic replacement. Differences in complication rate were not significant (*p* = 0.55). However, the rate of reintervention is higher in the CCS group (8.1%) than in the FNS group (2.85%).

## Discussion

Traditionally, the two most utilized implants for synthesizing femoral neck fractures are the dynamic hip screw (DHS) and cannulated compression screws (CCS). DHS offers advantages such as increased resistance to shear forces, particularly in cases of comminution or high Pauwels' angle, and enhanced stability in osteoporotic bone. On the other hand, CCS presents benefits including minimal invasiveness, rotational stability, and decreased surgical duration. In this context, the development of the new femoral neck system (FNS) aims to combine the relative advantages of the existing devices. The few existing biomechanical studies generally suggest the mechanical superiority of FNS over CCS. For instance, Stoffel et al. [16] demonstrated comparable outcomes between FNS and DHS, both of which surpassed CCS in countering varus collapse and femoral neck shortening in anatomical models of unstable femoral neck fractures. Additionally, Schopper et al. [17] concluded that FNS exhibits mechanical superiority over Hansson pins, with this advantage being less affected by potential inaccuracies in device positioning compared to the use of the aforementioned pins. Operative time holds significant importance, particularly when considering frail patients, as prolonged operative durations are associated with increased blood loss, extended anesthesia duration, elevated risk of infection, and a higher incidence of postoperative complications. Although, the findings on operative times between FNS and CCS vary in the literature. Some studies have indicated significantly shorter operative times with FNS [18–20], while others [11, 21] have shown shorter surgical durations with cannulated screws or no significant difference [22]. Our study revealed a significantly shorter surgical time in the FNS group. All orthopedic surgeon at our hospital had more experience with CCS than with FNS. The positive outcome regarding operative time emphasizes the simplicity of the positioning technique for FNS. The duration of hospitalization is undoubtedly a crucial consideration due to its impact on the risk of nosocomial infections and its direct correlation with healthcare expenditure. Indeed, various factors can impact the duration of hospitalization. Often, patients are required to remain in the hospital due to challenges in family logistical challenges or await transfer to rehabilitation or long-term care facilities. In our study, the hospital stay was significantly shorter in the CCS group. However, it is challenging for us to definitively ascertain whether this difference is solely attributable to device characteristics or if other variables also play a role. Anemia has been identified as a negative prognostic factor for short-term survival after proximal femur fracture. In our study, we investigated the variation in blood hemoglobin levels before and after surgery, representing intraoperative bleeding. However, no significant difference was observed. It is important to note that patients who underwent transfusions were excluded from the calculation. This finding seems to contradict what is described in the literature [11, 23], which suggests that unlike percutaneous screw introduction, the need for a 3–4 cm incision during the FNS technique would inevitably lead to greater blood loss. Indeed, preoperative bleeding associated with the fracture may act as a confounding factor regarding the actual loss attributable to surgery. Additionally, in our study, we did not account for any intraoperative or perioperative fluid infusion, which represents another confounding parameter due to its potential to induce hemodilution.

It is widely recognized that proximal femur fractures often result in varying degrees of permanent loss of function at the hip joint, particularly among elderly patients. One significant observation is the manifestation of a limp while walking: The femoral neck shortening due to impaction at the fracture site results in decreased lever arm of the abductor muscles at the greater trochanter, leading to their weakness and subsequent pain. In this study, contrary to findings in the existing literature [16, 18, 22], no superiority of FNS compared with CCS in preventing femoral neck shortening was observed. This finding suggests that the appropriate placement of the three CCSs in an inverted triangle configuration can ensure sufficient fracture stability and prevent femoral neck shortening comparably to FNS. While there are no differences between the two groups regarding the development of femoral neck shortening and complications, it is crucial to highlight that complications arising in patients with CCS lead to a higher proportion requiring reoperation.

The Harris Hip Score is a critical measure for assessing the clinical impact of internal fixation for femoral neck fractures. It encompasses various factors including bone healing, osteonecrosis of the femoral head, internal fixation failure, femoral neck shortening, and complications. A higher Harris Hip Score indicates better hip function. In this study, the HHS of patients treated by FNS is higher than that of patients treated by CCS. This outcome could be attributed not only to the reduced incidence of femoral head necrosis, reintervention rate, and implant failure but also to the distinct rehabilitation protocols recommended for patients. Specifically, we tend to adopt a more conservative approach with CCS group, advising them to commence loading on the operated limb one-month post-surgery. Conversely, for patients treated with FNS, we recommend earlier partial weight-bearing. As a result, the FNS group undoubtedly experiences a swifter and more favorable recovery of hip function. Limitations of our study include its retrospective and nonrandomized nature. Additionally, the sample size was relatively small. Furthermore, our study was constrained by limited preoperative data, leading us to focus solely on intraoperative and final follow-up data for analysis. Finally, the surgeries were performed by different trauma surgeons.

## Conclusion

The FNS does not appear superior to CCS in terms of femoral neck shortening, length of stay hospitalization, complication rate, and reduction in hemoglobin levels. FNS appears superior to CCS in terms of HHS, operation time, and reoperation rate.

## References

[1] Sing CW, Lin TC, Bartholomew S, Bell JS, Bennett C, Beyene K, Bosco-Levy P, Bradbury BD, Chan AHY, Chandran M, Cooper C, de Ridder M, Doyon CY, Droz-Perroteau C, Ganesan G, Hartikainen S, Ilomaki J, Jeong HE, Kiel DP, Kubota K, Lai EC, Lange JL, Lewiecki EM, Lin J, Liu J, Maskell J, de Abreu MM, O’Kelly J, Ooba N, Pedersen AB, Prats-Uribe A, Prieto-Alhambra D, Qin SX, Shin JY, Sørensen HT, Tan KB, Thomas T, Tolppanen AM, Verhamme KMC, Wang GH, Watcharathanakij S, Wood SJ, Cheung CL, Wong ICK (2023) Global epidemiology of hip fractures: secular trends in incidence rate, post-fracture treatment, and all-cause mortality. J Bone Miner Res 38(8):1064–1075. 10.1002/jbmr.4821 37118993 10.1002/jbmr.4821

[2] GBD 2015 Tobacco Collaborators (2017) Smoking prevalence and attributable disease burden in 195 countries and territories, 1990–2015: a systematic analysis from the Global Burden of Disease Study 2015. Lancet 389(10082):1885–906 28390697 10.1016/S0140-6736(17)30819-X PMC5439023

[3] GBD 2015 Obesity Collaborators, Afshin A, Forouzanfar MH, Reitsma MB et al (2017) Health effects of overweight and obesity in 195 countries over 25 years. N Engl J Med 377(1):13–27. 10.1056/NEJMoa1614362 28604169 10.1056/NEJMoa1614362 PMC5477817

[4] Zhou W, Langsetmo L, Berger C et al (2013) Longitudinal changes in calcium and vitamin d intakes and relationship to bone mineral density in a prospective population-based study: the canadian multicentre osteoporosis study (camos). J Musculoskelet Neuronal Interact 13(4):470–9 24292617 PMC5112013

[5] Lewiecki EM, Wright NC, Curtis JR, Siris E, Gagel RF, Saag KG, Singer AJ, Steven PM, Adler RA (2018) Hip fracture trends in the United States, 2002 to 2015. Osteoporos Int 29(3):717–722. 10.1007/s00198-017-4345-0 29282482 10.1007/s00198-017-4345-0

[6] World Health Organization (2011) Global health and aging. World Health Organization, Geneva

[7] Xu JL, Liang ZR, Xiong BL, Zou QZ, Lin TY, Yang P, Chen D, Zhang QW (2019) Risk factors associated with osteonecrosis of femoral head after internal fixation of femoral neck fracture: a systematic review and meta-analysis. BMC Musculoskelet Disord 20(1):632. 10.1186/s12891-019-2990-5.PMID:31884960;PMCID:PMC6935498 31884960 10.1186/s12891-019-2990-5.PMID:31884960;PMCID:PMC6935498 PMC6935498

[8] Brox WT, Roberts KC, Taksali S et al (2015) The american academy of orthopaedic surgeons evidence-based guideline on management of hip fractures in the elderly. J Bone Joint Surg Am 97(14):1196–1199. 10.2106/JBJS.O.00229.PMID:26178894;PMCID:PMC6948785 26178894 10.2106/JBJS.O.00229.PMID:26178894;PMCID:PMC6948785 PMC6948785

[9] Yan SG, Cui Y, Li D, Liu F, Hua X, Schmidutz F (2023) Femoral neck system versus three cannulated screws for fixation of femoral neck fractures in younger patients: a retrospective cohort study. J Invest Surg 36(1):2266752. 10.1080/08941939.2023.2266752 37813399 10.1080/08941939.2023.2266752

[10] Lin H, Lai C, Zhou Z, Wang C, Yu X (2023) Femoral Neck System vs. four cannulated screws in the treatment of Pauwels III femoral neck fracture. J Orthop Sci 6:1373–1378. 10.1016/j.jos.2022.09.006 10.1016/j.jos.2022.09.006 36229352

[11] Zhou Y, Li Z, Lao K, Wang Z, Zhang L, Dai S, Fan X (2023) Femoral neck system versus cannulated screws on treating femoral neck fracture: a meta-analysis and system review. Front Surg 10:1224559. 10.3389/fsurg.2023.1224559 37533744 10.3389/fsurg.2023.1224559 PMC10390772

[12] Gupta GK, Rai A, Mandal S, Rani S, Shekhar S, Halder S, Prasad P, Kumar A, Haque ZU (2022) Comparison of femoral neck system versus cannulated cancellous screws for the fixation of femoral neck fracture in young adults: a systematic review and meta-analysis. Cureus 14(11):e32011. 10.7759/cureus.32011 36589187 10.7759/cureus.32011 PMC9798663

[13] Rajnish RK, Srivastava A, Rathod PM, Haq RU, Aggarwal S, Kumar P, Dhammi IK, Dadra A (2022) Does the femoral neck system provide better outcomes compared to cannulated screws fixation for the management of femoral neck fracture in young adults? A systematic review of literature and meta-analysis. J Orthop 32:52–59. 10.1016/j.jor.2022.05.007 35601207 10.1016/j.jor.2022.05.007 PMC9118353

[14] Garden RS (1971) Malreduction and avascular necrosis in subcapital fractures of the femur. J Bone Joint Surg Br 53(2):183–197 5578215 10.1302/0301-620X.53B2.183

[15] Yin J, Zhu H, Gao Y, Zhang C (2019) Vascularized fibular grafting in treatment of femoral neck nonunion: a prognostic study based on long-term outcomes. J Bone Joint Surg Am 101(14):1294–1300. 10.2106/JBJS.18.01132 31318809 10.2106/JBJS.18.01132

[16] Stoffel K, Zderic I, Gras F, Sommer C, Eberli U, Mueller D, Oswald M, Gueorguiev B (2017) Biomechanical evaluation of the femoral neck system in unstable pauwels III femoral neck fractures: a comparison with the dynamic hip screw and cannulated screws. J Orthop Trauma 3:131–137. 10.1097/BOT.0000000000000739 10.1097/BOT.0000000000000739 27755333

[17] Schopper C, Zderic I, Menze J, Müller D, Rocci M, Knobe M, Shoda E, Richards G, Gueorguiev B, Stoffel K (2020) Higher stability and more predictive fixation with the femoral neck system versus hansson pins in femoral neck fractures pauwels II. J Orthop Translat 24:88–95. 10.1016/j.jot.2020.06.002 32775200 10.1016/j.jot.2020.06.002 PMC7387742

[18] Vazquez O, Gamulin A, Hannouche D, Belaieff W (2021) Osteosynthesis of non-displaced femoral neck fractures in the elderly population using the femoral neck system (FNS): short-term clinical and radiological outcomes. J Orthop Surg Res 16(1):477. 10.1186/s13018-021-02622-z 34348753 10.1186/s13018-021-02622-z PMC8336369

[19] Nibe Y, Matsumura T, Takahashi T, Kubo T, Matsumoto Y, Takeshita K (2022) A comparison between the femoral neck system and other implants for elderly patients with femoral neck fracture: a preliminary report of a newly developed implant. J Orthop Sci 27(4):876–880. 10.1016/j.jos.2021.04.016 34090779 10.1016/j.jos.2021.04.016

[20] Yang Y, Ma T, Zhang X, Luo X, Fan T, Wang Y (2022) Short-term effectiveness of femoral neck system in the treatment of femoral neck fracture. Zhongguo Xiu Fu Chong Jian Wai Ke Za Zhi 35(5):539–543. 10.7507/1002-1892.202012097 10.7507/1002-1892.202012097 PMC8175206 33998204

[21] Hu H, Cheng J, Feng M, Gao Z, Wu J, Lu S (2021) Clinical outcome of femoral neck system versus cannulated compression screws for fixation of femoral neck fracture in younger patients. J Orthop Surg Res 16(1):370. 10.1186/s13018-021-02517-z 34107990 10.1186/s13018-021-02517-z PMC8188789

[22] Lu Y, Huang Z, Xu Y, Huang Q, Ren C, Li M, Li Z, Sun L, Xue H, Zhang K, Wang Q, Ma T (2022) Femoral neck system versus cannulated screws for fixation of femoral neck fracture in young adults: a systematic review and meta-analysis. Am J Transl Res 14(8):5480–5490 36105033 PMC9452327

[23] Yeoh SC, Wu WT, Peng CH, Yao TK, Chang CM, Liu KL, Yu TC, Chen IH, Wang JH, Yeh KT (2024) Femoral neck system versus multiple cannulated screws for the fixation of Pauwels classification type II femoral neck fractures in older female patients with low bone mass. BMC Musculoskelet Disord 25(1):62. 10.1186/s12891-024-07179-6 38218794 10.1186/s12891-024-07179-6 PMC10787435

